# Theaflavin‐3,3´‐digallate increases the antibacterial activity of β‐lactam antibiotics by inhibiting metallo‐β‐lactamase activity

**DOI:** 10.1111/jcmm.14580

**Published:** 2019-08-08

**Authors:** Zihao Teng, Yan Guo, Xingqi Liu, Jian Zhang, Xiaodi Niu, Qinlei Yu, Xuming Deng, Jianfeng Wang

**Affiliations:** ^1^ Department of Respiratory Medicine The First Hospital of Jilin University Changchun China; ^2^ Key Laboratory of Zoonosis Research, Ministry of Education, Institute of Zoonosis, College of Veterinary Medicine Jilin University Changchun China; ^3^ Jilin Provincial Animal Disease Control Center Changchun China

**Keywords:** metallo‐β‐lactamase, *Staphylococcus aureus*, theaflavin‐3,3´‐digallate, β‐lactam antibiotic, β‐lactamase inhibitor

## Abstract

Metallo‐β‐lactamases (MBLs) are some of the best known β‐lactamases produced by common Gram‐positive and Gram‐negative pathogens and are crucial factors in the rise of bacterial resistance against β‐lactam antibiotics. Although many types of β‐lactamase inhibitors have been successfully developed and used in clinical settings, no MBL inhibitors have been identified to date. Nitrocefin, checkerboard and time‐kill assays were used to examine the enzyme behaviour in vitro. Molecular docking calculation, molecular dynamics simulation, calculation of the binding free energy and ligand‐residue interaction decomposition were used for mechanistic research. The behaviour of the enzymes in vivo was investigated by a mouse infection experiment. We showed that theaflavin‐3,3´‐digallate (TFDG), a natural compound lacking antibacterial activities, can inhibit the hydrolysis of MBLs. In the checkerboard and time‐kill assays, we observed a synergistic effect of TFDG with β‐lactam antibiotics against methicillin‐resistant *Staphylococcus aureus* BAA1717. Molecular dynamics simulations were used to identify the mechanism of the inhibition of MBLs by TFDG, and we observed that the hydrolysis activity of the MBLs was restricted by the binding of TFDG to Gln242 and Ser369. Furthermore, the combination of TFDG with β‐lactam antibiotics showed effective protection in a mouse *Staphylococcus aureus* pneumonia model. These findings suggest that TFDG can effectively inhibit the hydrolysis activity of MBLs and enhance the antibacterial activity of β‐lactam antibiotics against pathogens in vitro and in vivo.

## 1 INTRODUCTION

β‐Lactam antibiotics are one of the earliest and most used antimicrobial agents for the treatment of bacterial infections and have saved innumerable lives. However, the abuse of antibiotics has also caused a variety of serious antibiotic resistance problems. Β‐Lactamase is the primary cause of resistance to β‐lactam antibiotics, which has evolved thousands of variants and is widely distributed in various Gram‐positive and Gram‐negative pathogens.^1^, ^2^ Ambler has categorized β‐lactamases into classes A to D according to amino acid sequence homology, with class A, C and D β‐lactamases being serine‐dependent enzymes while class B β‐lactamases (metallo‐β‐lactamases, MBLs) are zinc‐dependent enzymes.^3^ To prolong the usefulness of β‐lactam antibiotics, β‐lactamase inhibitors have been being developed for nearly 40 years.^4^ Presently, numerous types of inhibitors targeting classes A, C and D enzymes are used in clinical treatment settings, including sulbactam, avibactam and clavulanic acid, within impressive treatment outcomes. However, while no MBL inhibitors have been successfully developed, the rate at which MBLs are expressed by pathogens, such as *Staphylococcus aureus* (*S aureus*), has risen to 20%‐30%, resulting from the dissemination of resistant plasmids among strains and the increasing application of inhibitors targeting other β‐lactamases.^5^ Thus, it is urgent to develop new MBL inhibitors to combat the increase in bacterial resistance to β‐lactam antibiotics.

*S aureus* is a common Gram‐positive pathogen that is among the major causes of many infectious diseases, including pneumonia, sepsis and endocarditis.^6^ *S aureus* infections have always been difficult to treat because of the occurrence of antibiotic‐resistant strains, especially methicillin‐resistant *Staphylococcus aureus* (MRSA), which represents a great threat to human health. Since the first use of antimicrobials, most antibiotics have become drastically less effective due to the continuous development of resistance mechanisms, even to the antibiotics vancomycin and linazolamide, which were once considered the last treatment options for MRSA infections.^7^ *S aureus* was the first known pathogen to develop resistance to β‐lactam antibiotics by secreting β‐lactamase to break the amide bond of β‐lactam rings.^8^ Since the use of fifth‐generation cephalosporin, penicillin‐binding protein (PBP) mutation, one of the reasons for the resistance of *S aureus*, may no longer be the major cause of induced resistance. However, β‐lactamase is still an unsolved resistance factor in clinical practice. Studies have shown that 95% of clinical *S aureus* isolates can express different types of β‐lactamases, which are the primary cause of β‐lactam antibiotic resistance.^6^

**Figure 1:**
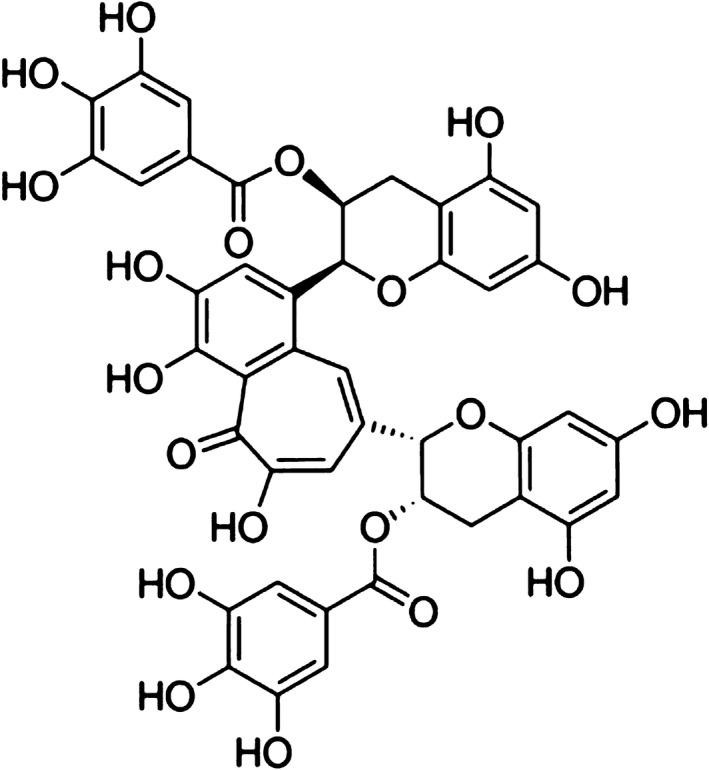
Chemical structure of TFDG

Theaflavin‐3,3´‐digallate (TFDG, Figure 1) is one of the most important active natural compounds in theaflavin and is believed to play a primary role in the antibacterial, antitumour, anti‐inflammatory, free radical scavenging and lipid‐lowering activities of black tea.^9^, ^10^, ^11^, ^12^, ^13^ In this study, TFDG was identified as an effective MBL inhibitor using a nitrocefin assay, and we demonstrated the protective capability of TFDG combined with β‐lactam antibiotic usage against pneumonia caused by MRSA.

## 2 MATERIALS AND METHODS

### 2.1 Bacteria and chemicals

The *S aureus* strains BAA1717 and ATCC 29213 and the NDM‐1‐producing *E coli* isolate were used in this study: the NDM‐1 producing strain was isolated in a previous study,^14^ and TEM‐1 was purified from pET‐21a plasmid, as described in Table 1. TFDG and antibiotics were obtained from Dalian Meilun Biotechnology Co., Ltd. TFDG was dissolved in dimethyl sulphoxide (DMSO), and the antibiotics used in this study were prepared in sterile water.

**Table 1:** The description of β‐lactamases used in this study

Protein	Origin	EC number	Protein ID
β‐lactamase N1	*S aureus* BAA1717	3.5.2.6	ABX29221.1
BAA1717‐BLA‐2	*S aureus* BAA1717	3.5.2.6	ABX28068.1
NDM‐1	*E coli* isolate	3.5.2.6	

### 2.2 Nitrocefin assay

In this study, we amplified and expressed MBL variants (β‐lactamase N1, BAA1717‐BLA‐2 and NDM‐1) and TEM‐1 (class A β‐lactamase) from *S aureus*, *E coli* strains and pET‐21a plasmid for use in further experiments. The protein‐coding genes were cloned into pET‐21a using the primers shown in Table 2. The combined plasmids were tested by next‐generation sequencing and then transformed into BL21 (DE3) competent cells. The BL21 (DE3) cells with the recombinant gene were cultured to OD_600 nm_ = 0.6 at 37°C and then were cultured with 0.2 mmol/L IPTG with shaking at 16°C for 8 hours. After centrifugation, the bacteria were resuspended in sterile phosphate buffer (pH = 7.2) and broken by ultrasound in an ice bath. Then, the mixtures were centrifuged at 4°C, and the supernatants were collected for the subsequent protein purification as described by *Liu*.^15^ The Gln242Ala and Ser369Ala mutants of β‐lactamase N1 were expressed and purified as described above, and the primers used for mutation are shown in Table 2.

**Table 2:** Primer sequences used in this study

Primer	Sequence (5′‐3′)
*bla* ‐1717‐N1‐F	gcgcggatccATGAGTTTAATAAAGAAAAAGAATAAAG
*bla* ‐1717‐N1‐R	gcgcctcgagTTAAATTTCAGAAATTACTGGAATAAT
*bla* ‐1717‐2‐F	gcgcggatccATGAGCCGCTTGATACGCATG
*bla* ‐1717‐2‐R	gcgcctcgagTTATATTGTATATATTGGCGTTGGAATAG
*bla* –ndm‐F	gcgcggatccGTGCTGGTGGTCGATAC
*Bla* –ndm‐R	gcgcctcgagTCAGCGCAGCTTGTCG
*bla* ‐242‐F	GCTTCGAACTTTATACGTATTGCGCAAGTTTTAAATATTGCTAG
*bla* ‐242‐R	CTAGCAATATTTAAAACTTGCGCAATACGTATAAAGTTCGAAGC
*bla* ‐369‐F	GATTCATGCTTCAGCTCATGGTTGCATGG
*bla* ‐369‐R	CCATGCAACCATGAGCTGAAGCATGAATC

A nitrocefin assay was used for the screening of potential effective inhibitors and further the determination of the inhibitory effect of TFDG on the hydrolysis activities of MBLs. Nitrocefin serves as an indicator whose colour changes from yellow to red with increased hydrolysis. β‐Lactamase N1 (500 ng/mL), BAA1717‐BLA‐2 (500 ng/mL) and NDM‐1 (250 ng/mL) were incubated with various concentrations of TFDG (0, 4, 8, 16 and 32 μg/mL) in phosphate buffer (pH = 7.2) at 37°C for 5 minutes, and then, 50 μg/mL of nitrocefin was added to the mixture. After 10 minutes of incubation, the samples were read at OD_492 nm_ to determine the level of nitrocefin hydrolysis. Additionally, the inhibitory effect of TFDG against β‐lactamase N1 in the presence of excess zinc ion was further evaluated as described above.

### 2.3 Synergy evaluation

The fractional inhibitory concentration (FIC) was evaluated by checkerboard assays using the protocol and calculation formula described by *Novy*.^16^ In this assay, BAA1717 was cultured to OD_600 nm_ = 0.1 at 37°C and then diluted to 5 × 10^5^ CFUs/mL in trypticase soy broth medium (TSB). The minimum inhibitory concentrations (MICs) of TFDG and β‐lactam antibiotics in combination and the MICs of TFDG and of β‐lactam antibiotics alone were tested with three replications at 37°C. The synergistic effects were evaluated by determining the fractional inhibitory concentration index (FICI), which was interpreted according to the European Committee on Antimicrobial Susceptibility Testing as follows: FICI ≤ 0.5 denotes synergy; 0.5 < FICI ≤ 4 denotes no interaction; and FICI > 4 denotes antagonism.

To determine whether the synergistic effect is related to other resistant mechanisms, we also tested TFDG and cephalothin in combination against MSSA *S aureus* strain ATCC 29213 (which was reported to express class A and class C β‐lactamases). In addition, tetracycline and erythromycin were separately combined with TFDG against BAA1717 to determine the specificity of TFDG with regard to β‐lactam antibiotics.

### 2.4 Time‐kill assay

A time‐kill assay was performed to further determine the synergistic effect of TFDG with β‐lactam antibiotics according to *Alexandre's* method.^17^ In this assay, BAA1717 was used as the experimental strain. The concentrations used were 32 μg/mL, and cephalothin was used at 2 μg/mL. BAA1717 was grown at 37°C in TSB with shaking to an OD_600 nm_ of 0.1 and diluted to 5 × 10^5^ CFUs/mL and then statically incubated with various combinations of drugs for 0, 1, 2, 4 and 6 hours at 37°C. Subsequently, each sample was diluted in sterile phosphate buffer (pH = 7.2) and plated on TBS agar plates.

### 2.5 Structural modelling of β‐lactamase N1 and molecular docking calculation

β‐Lactamase N1 (isolated from BAA1717) was used to investigate the binding mode between TFDG and MBLs. We used homology‐based modelling to gain insights into the molecular mechanism of action of TFDG. Because the structure of monomeric β‐lactamase N1 is not available, we used the crystal structure information of its homolog RNase J1 (PDB code: 3ZQ4) to infer the structure of β‐lactamase N1 by using MODELLER (version 9.9). This program optimizes the structure of the homology models by minimizing a global probability density function that integrates the stereochemical parameters and homology‐derived restraints.^18^ The best model was selected based on its DOPE score and subjected to a further 1000 ns molecular dynamics (MD) simulation using the Gromacs 5.1 software package.^19^ The geometry of TFDG was optimized at the B3LYP/6‐31G* level using the program Gaussian 09.^20^

To obtain the initial structure of the β‐lactamase N1‐TFDG complex for subsequent MD simulation, a standard docking procedure for a rigid protein and a flexible ligand was performed using AutoDock 4.^21^, ^22^ The Lamarckian genetic algorithm (LGA) was applied in the docking calculations. During the simulation, all of the torsional bonds of the drug were allowed to rotate freely, whereas the β‐lactamase N1 molecule was held rigid. Next, the polar hydrogen atoms were added to β‐lactamase N1 using the AutoDock tools, and Kollman united atom partial charges were then assigned.^23^ A total of 150 independent runs were carried out with the maximum number of energy evaluations set to 2.5 × 10^7^ and using a population size of 300. A grid box (28 × 22 × 32) with spacing of 0.1nm was created and centred on the mass centre of the ligand. Energy grid maps for all possible ligand atom types were generated using AutoGrid 4 before performing the docking.

### 2.6 Molecular dynamics simulation

The Gromacs 5.1 software package was used for all simulations, while the analysis of the trajectories was performed with the Amber ff99sb force field and the TIP3P water model.^19^ The β‐lactamase N1‐TFDG systems were first energy relaxed with 2000 steps of steepest‐descent energy minimization followed by another 2000 steps of conjugate‐gradient energy minimization. The system was then equilibrated by a 500 ps MD run with position restraints on the protein and ligand to allow relaxation of the solvent molecules. The first equilibration run was followed by a 200 ns MD run without position restraints on the solute. The first 20 ns of the trajectory were not used in the subsequent analysis to minimize convergence artefacts. The equilibration of the trajectory was assessed by monitoring the equilibration of specific values, such as the root‐mean‐square deviation (RMSD) with respect to the initial structure, the internal protein energy and the fluctuations calculated for different time intervals. The electrostatic term was described with the particle mesh Ewald algorithm. The LINCS algorithm was used to constrain all bond lengths.^24^ For the water molecules, the SETTLE algorithm was used. A dielectric permittivity of *ε* = 1 and a time step of 2 fs were used.^25^ All atoms were given an initial velocity obtained from a Maxwellian distribution at the desired initial temperature of 300 K. The density of the system was adjusted during the first equilibration runs under *NPT* conditions by weak coupling to a constant‐pressure bath (*P*_0_ = 1 bar, coupling time *τ_P_* = 0.5 ps).^26^ In all simulations, the temperature was maintained close to the intended values by weak coupling to an external temperature bath with a coupling constant of 0.1 ps. The proteins and the rest of the system were coupled separately with the temperature bath. The structural cluster analysis was carried out using the method described by Daura and coworkers with a cut‐off of 0.25 nm.^25^

The TFDG parameters were estimated with the antechamber programs and AM1‐BCC partial atomic charges from the Amber suite of programs.^26^, ^27^ The trajectories were analysed using the PyMOL and Gromacs analysis tools.

### 2.7 Calculation of binding free energies

The binding free energies were calculated using the MM‐GBSA approach in the Amber 10 package. We chose a total number of 200 snapshots distributed evenly throughout the last 50 ns on the MD trajectory with an interval of 10 ps. The MM‐GBSA method can be conceptually summarized as follows:

(1) $$\Delta G_{\text{bind}}\;=\;\Delta G_{\text{comple}}\;-\;\left[\Delta G_{\text{protein}}\;+\;\Delta G_{\text{TFDG}}\right]$$

(2) $$\Delta G_{\text{bind}}=\;\Delta H\;-\;T\Delta S$$

where Δ*H* of the system consists of the enthalpy changes in the gas phase upon complex formation (Δ*E*_MM_) and the solvated free energy contribution (Δ*G*_sol_), while *–T*Δ*S* refers to the contribution of entropy to the binding. Equation (2) can then be approximated as shown below:

(3) $$\Delta G_{\text{bind}}=\;\Delta E_{\text{MM}}+\Delta G_{\text{sol}}-\;T\Delta S$$

where Δ*E*_MM _is the summation of the van der Waals (Δ*E*_vdw_) and the electrostatic (Δ*E*_ele_) interaction energies.

(4) $$\Delta E_{\text{MM}}=\Delta E_{\text{vdw}}+\Delta E_{\text{ele}}$$

In addition, Δ*G*_sol_, which denotes the solvation free energy, can be computed as the summation of an electrostatic component (Δ*G*_ele,sol_) and a nonpolar component (Δ*G*_nonpolar,sol_), as shown in Equation (5):

(5) $$\Delta G_{\text{sol}}=\Delta G_{\text{ele},\text{sol}}+\Delta G_{\text{nonpolar},\text{sol}}$$

### 2.8 Ligand‐residue interaction decomposition

The interactions between TFDG and all residues of β‐lactamase N1 were analysed using the MM‐GBSA decomposition process applied in the MM‐GBSA module in Amber 10. The binding of each TFDG‐residue pair includes three terms: the van der Waals contribution (Δ*E*_vdw_), the electrostatic contribution (Δ*E*_ele_) and the solvation contribution (Δ*E*_sol_). All energy components were calculated using the same snapshots as the free energy calculation.

### 2.9 Mouse model of *S aureus* pneumonia

Six‐ to eight‐week‐old female BALB/c mice were supplied by the Jilin University Experimental Animal Center and were fed and handled according to the standards approved by the Animal Welfare and Research Ethics Committee of Jilin University.

BAA1717 was used as an experimental strain and was cultured in TSB to an OD_600 nm_ of 0.8 at 37°C. After the culture, the bacteria were centrifuged at 3000 g for 3 minutes, resuspended in phosphate buffer (pH = 7.2) and quantified at OD_600 nm_. The mice were narcotized with ether and nasally infected with 20 μL (approximately 1.5 × 10^8^ CFUs per 20 μL) of a bacterial suspension, divided into four groups: the cephalothin (15 mg/kg, approximately one quarter of the normal dose) group, the TFDG (50 mg/kg) group, the combination (15 mg/kg of cephalothin and 50 mg/kg of TFDG) group and the control (DMSO) group. Approximately 2 hours after infection, the mice were treated with the corresponding drugs by hypodermic injection at 12‐hours intervals. Each group consisted of 10 mice. TFDG and cephalothin were injected separately in the combination group to prevent the possible precipitation of TFDG.

The mortality rate of the infected mice was monitored daily. To evaluate the pathological changes, the lungs were placed in 4% formalin, stained with haematoxylin and eosin, and then observed using a light microscope. To assess the colonization of bacteria, the lungs were first weighed and then grounded in PBS containing 2% Triton and plated on TSB agar plates. To evaluate the presence of pneumonedema, the wet‐dry weight ratios of the removed lungs were determined by weighing the lungs before (wet weight) and after (dry weight) being dried in an 80°C air oven.

### 2.10 Statistical analysis

The significance levels of hydrolytic activity, bacterial counts and lung wet‐dry ratios were calculated using two‐tailed Student's *t* tests, with * indicating *P* < .05 and ** indicating *P* < .01 compared with the control group.

## 3 RESULTS

### 3.1 TFDG inhibits the hydrolytic activity of MBLs

**Figure 2:**
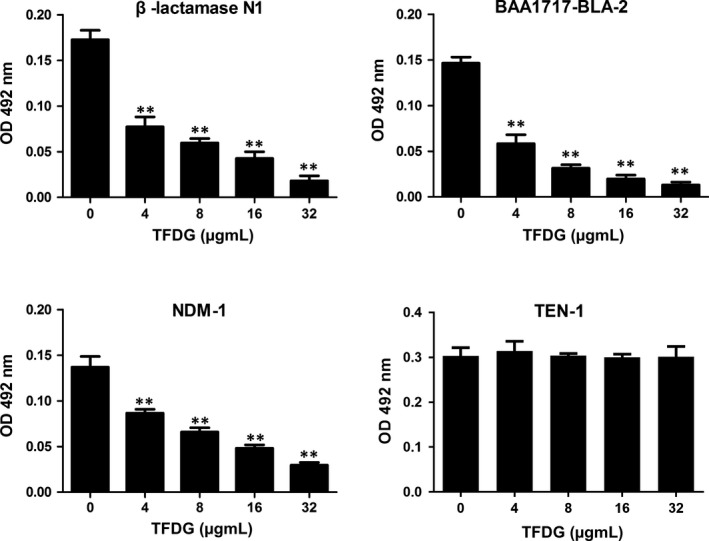
TFDG inhibits the hydrolytic activity of MBLs. Nitrocefin was incubated with four β‐lactamase variants pretreated with various concentrations of 32 μg/mL TFDG, after which the samples were measured at OD_492 nm_. Bars represent the standard deviation (** indicates *P* < .01 compared with the control group; two‐tailed Student's test)

In the nitrocefin assay, purified β‐lactamases were incubated with nitrocefin and TFDG. When TFDG was added at 4 μg/mL, the hydrolysis of nitrocefin in the MBL groups were significantly inhibited by TFDG in a dose‐dependent manner, and no inhibitory effect was detected in the TEM‐1 group (Figure 2). The IC_50_ values of TFDG against β‐lactamase N1, BAA1717‐BLA‐2 and NDM‐1 were 5.82 μg/mL, 4.54 μg/mL and 8.75 μg/mL. Thus, these results indicated that TFDG may be a potential inhibitor of MBLs.

### 3.2 TFDG increases the bactericidal ability of β‐lactams against MRSA

Checkerboard assays were used to investigate the synergistic effects of TFDG and antibiotics against BAA1717. BAA1717 is a typical MRSA strain that can efficiently express multiple variants of MBLs. The MICs of antibiotics either alone or in combination with TFDG are shown in Table 3, and the FICI values of the combinations are summarized in Table 3. In this assay, all the β‐lactam antibiotics assayed were insensitive to MRSA, including penicillin antibiotics, first‐generation cephalosporins, second‐generation cephalosporins and third‐generation cephalosporins. However, the addition of TFDG resulted in a fourfold or eightfold (FICI = 0.313 or 0.188) reduction in the MICs of β‐lactam antibiotics against BAA1717, while TFDG alone exhibited only weak antibacterial activity. The calculated FICI results also showed significant synergistic effects of TFDG with antibiotics.

**Table 3:** The MICs and FICI of β‐lactam antibiotics combined with TFDG

BAA1717	MIC (μg/mL)										
	TFDG	A	B	C	D	E	F	G	H	I	J
	512	256	256	16	16	128	64	64	128	128	64
TFDG (32μg/mL)		32	32	4	4	16	16	8	16	32	8
FIC index											
BAA1717		0.188	0.188	0.313	0.313	0.188	0.313	0.188	0.188	0.313	0.188

Note

Penicillins: A, penicillin; B, ampicillin. First‐generation cephalosporins: C, cephalothin; D, cefazolin; E, cefradine. Second‐generation cephalosporins: F, cefuroxime; G. cefaclor. Third‐generation cephalosporins: H, cefoperazone; I, ceftazidime; J, ceftriaxone.

To estimate whether the synergistic effect is related to other resistance mechanisms, we also tested TFDG and cephalothin in combination against MSSA *S aureus* strain ATCC 29213 (which was reported to express class A and class C β‐lactamases). The results showed that the MIC of cephalothin against ATCC 29213 was 0.25 μg/mL both with and without 32 μg/mL of TFDG (not shown in the table). On the other hand, tetracycline and erythromycin were separately combined with TFDG against BAA1717 to determine the specificity of TFDG for β‐lactam antibiotics. The results showed that TFDG (32 μg/mL) cannot reduce the MICs of tetracycline (16 μg/mL) and erythromycin (2 μg/mL).

**Figure 3:**
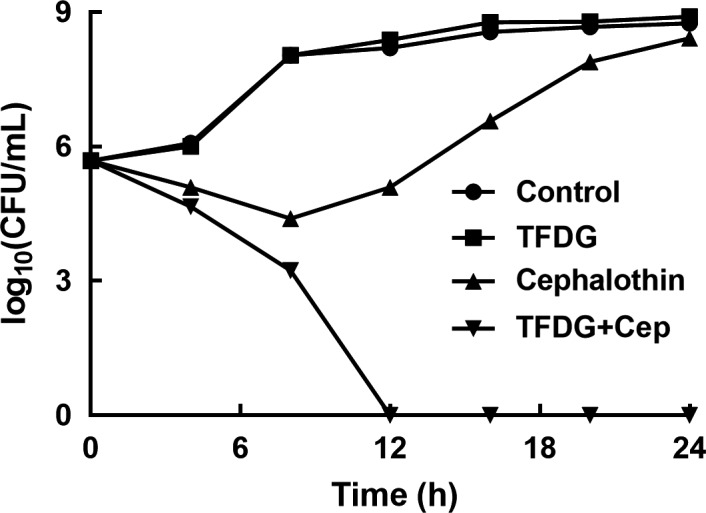
Time‐kill curves of BAA1717 cultured with different combinations of compounds. BAA1717 was incubated with different combinations of compounds and plated on TBS agar plates after dilution in sterile phosphate buffer (pH = 7.2). Control: BAA1717 without any treatment; TFDG: BAA1717 was treated with 32 μg/mL TFDG; Cephalothin: BAA1717 was treated with 2 μg/mL cephalothin; TFDG + Cep: BAA1717 was treated with 32 μg/mL TFDG and 2 μg/mL cephalothin

Time‐kill assays were performed to further evaluate the observed antibacterial effect. As expected, the growth of BAA1717 was significantly inhibited by the combination of TFDG and cephalothin, and the bacteria were almost killed at 12 hours (Figure 3).

Taken together, our results established that TFDG increased the bactericidal ability of β‐lactams against MRSA in vitro.

### 3.3 The binding mode of β‐lactamase N1 with TFDG

**Figure 4:**
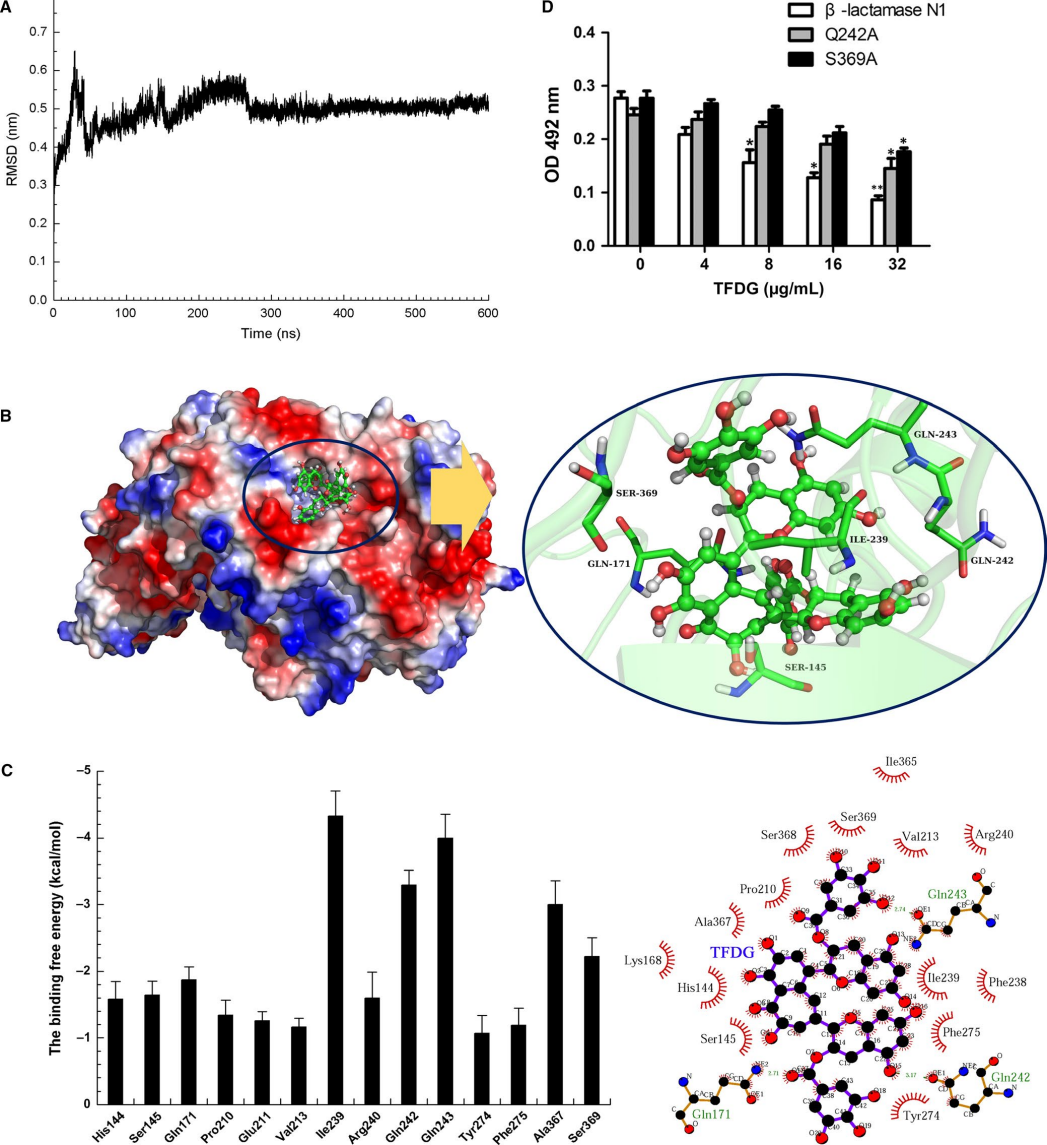
Identification of the mechanism of TFDG against β‐lactamase N1. A, The RMSD of backbone atoms of the protein in the β‐lactamase N1‐TFDG complex. B, The predicted binding mode of TFDG to β‐lactamase N1. C, The energy contributions from selected residues. D, The influence of TFDG on the hydrolytic activity of β‐lactamase N1 and its two mutants. Nitrocefin was incubated with MBLs and various concentrations of TFDG, after which the samples were measured at OD_492 nm_. Bars represent the standard deviation (** indicates *P* < .01 compared with the control group; two‐tailed Student's test)

The preferential binding mode of β‐lactamase N1 with TFDG was determined by 600 ns MD simulations based on the docking results. To explore the dynamic stability of the models and to ensure the rationality of the sampling strategy, the RMSD values of the protein backbone based on the starting structure over the course of the simulation were calculated and are plotted in Figure 4A. The results showed that the protein structures of all the systems were stabilized during the simulations.

In the simulation, TFDG is a ligand that can bind to β‐lactamase N1 via intermolecular interactions. Over the time course of the simulation, TFDG localizes to the ‘active’ region of β‐lactamase N1. The predicted binding mode of TFDG to β‐lactamase N1 is illustrated in Figure 4B, and the electrostatic potentials of the residues around the binding site were mapped using APBS software.^28^ In detail, the binding model of TFDG to the active region of β‐lactamase N1 (Figure 4B) revealed that the carbonyl and amino groups of Gln243 can form two hydrogen bonds with the hydroxyl moiety of TFDG. In addition, the residues Ile239, Gln242 and Ser369 were proximal to TFDG, suggesting that TFDG can form strong interactions with these residues.

The results described above indicated that the stabilization at the binding cavity of β‐lactamase N1 in this complex was mostly due to residues Ile239, Gln242, Gln243 and Ser369, as shown in Figure 4B.

### 3.4 Identification of the binding site in the β‐lactamase N1‐TFDG complex

To obtain additional details regarding the residues surrounding the binding site and their contribution to the whole system, the electrostatic, van der Waals, solvation and total contributions of the residues to the binding free energy were calculated using the molecular mechanics generalized Born surface area (MM‐GBSA) method.^29^, ^30^ The calculation was performed over the 600 MD snapshots taken from the last 100 ns of the simulation. The energy contributions from the selected residues are summarized in Figure 4C. The results showed that in the β‐lactamase N1‐TFDG complex, Ile239 had the strongest binding energy contribution with a ΔE of ≤ −4.326 kcal/mol. In fact, Ile239 was close to the 4H‐chromen‐4‐one moiety of TFDG, and a strong hydrophobic interaction between the two moieties was observed (Figure 4C). Furthermore, residue Gln242, with a ΔE of ≤ −3.294 kcal/mol, exhibited strong van der Waals interactions with the ligand because of the close proximity between the residue and TFDG. Moreover, residue Gln243, with a ΔE of ≤−3.996 kcal/mol, also exhibited strong interactions with the ligand due to the formation of hydrogen bonds with TFDG. In addition, Ser369 had a ΔE of <−2.00 kcal/mol. Thus, residues Ile239, Gln242, Gln243 and Ser369 were observed to play key roles in the binding of β‐lactamase N1 with TFDG. Due to the binding of TFDG with the active site (residues Ile239, Gln242, Gln243 and Ser369), the activity of β‐lactamase N1 was inhibited.

**Figure 5:**
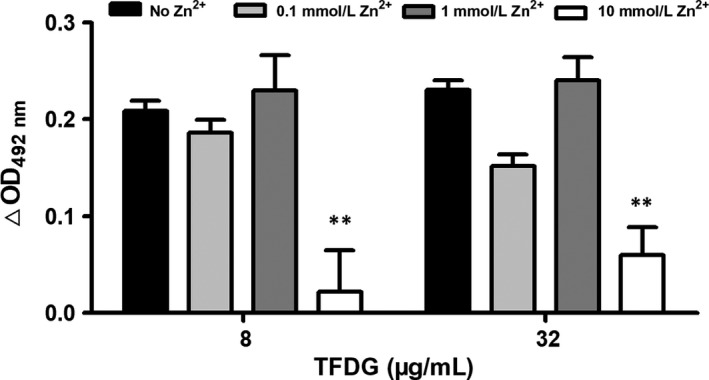
Excess zinc ion hinders TFDG‐mediated inhibition of β‐lactamase N1 activity. The activity of TFDG‐treated β‐lactamase N1 in the presence of excess zinc ion was determined as described in Figure 2. △OD_492 nm_ = OD_492 nm_ of the sample without TFDG‐ OD_492 nm_ of the sample with the indicated concentration of TFDG (** indicates *P* < .01 compared with the control group)

To investigate the importance of Ile239, Gln242, Gln243 and Ser369 in the binding of β‐lactamase N1 and TFDG, we mutated these amino acids to alanine and tested the resulting proteins in a nitrocefin assay. As shown in Figure 4D, the inhibition of β‐lactamase N1 by TFDG was significantly higher than that observed for the Gln242Ala (Q242A) and Ser369Ala (S369A) mutants and the IC_50_ values of TFDG against Q242A and S369A were 28.91 μg/mL and 30.13 μg/mL. While no remarkable difference was observed for Ile239Ala and Gln243Ala (not shown). However, the inhibitory effect of TFDG against β‐lactamase N1 activity was hindered in the presence of excess zinc ion (Figure 5).

### 3.5 The combination of TFDG and cephalothin protects mice from *S aureus* pneumonia

**Figure 6:**
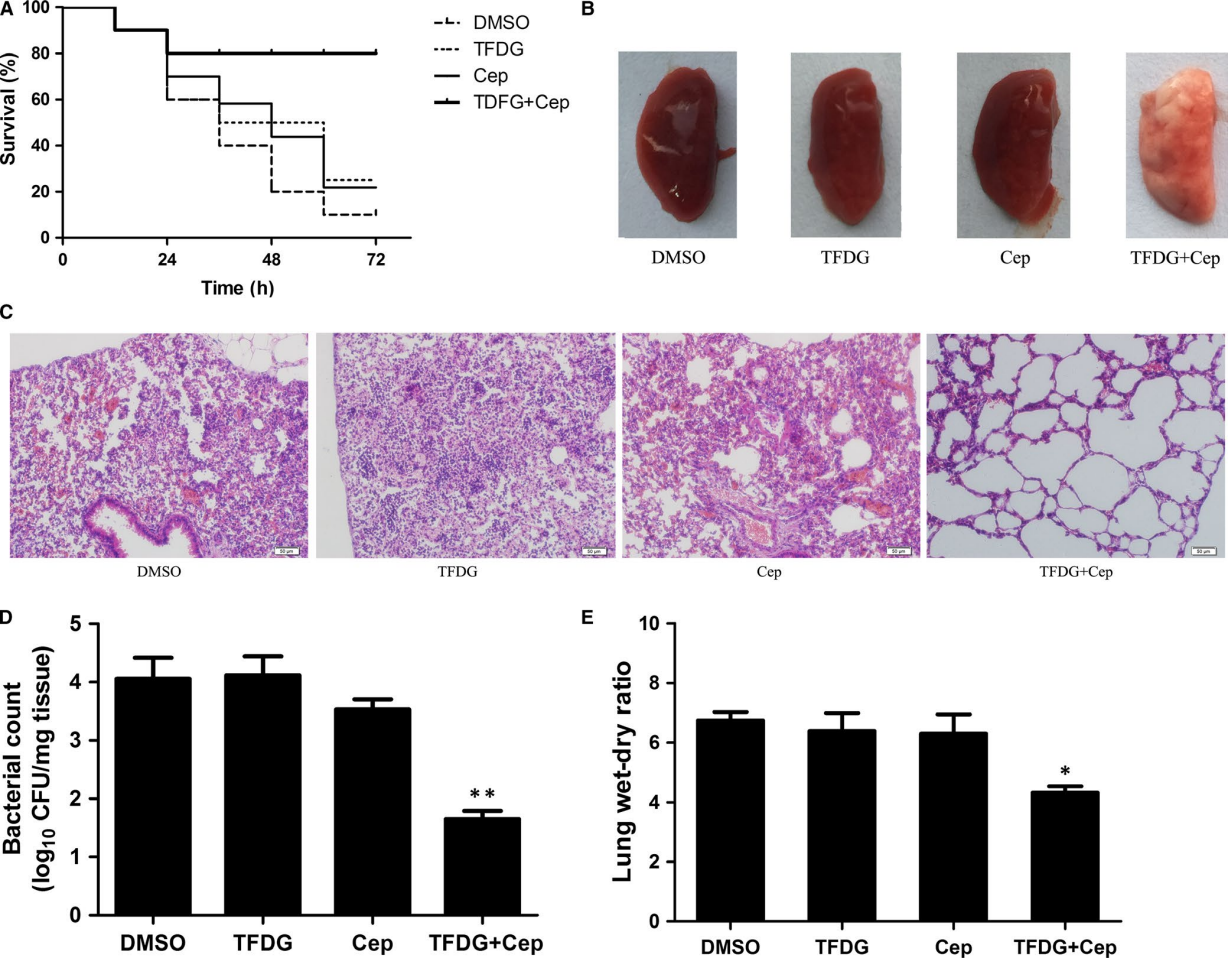
The combination of TFDG and a β‐lactam antibiotic protects mice from BAA1717 pneumonia. A, The combination of TFDG and a β‐lactam antibiotic protects infected mice from BAA1717 mortality. Kaplan‐Meier survival estimates were used for the mortality rate at 72 h. B, Pathological observation by naked eye and (C) light microscopy. D, The combination influences the colonization of BAA1717 in the lungs. E, The influence of the combination on the lung wet/dry weight ratio in BAA1717‐infected mice. Data are expressed as the means ± SD of three independent experiments. (* indicates *P* < .05 and ** indicates *P* < .01 compared with the control group; two‐tailed Student's test)

The FICI values and time‐kill assay results demonstrated that TFDG could increase the bactericidal ability of β‐lactams against MRSA in vitro. Based on these findings, we further assessed the protective effects of TFDG combined with cephalothin in a mouse pneumonia model. BALB/c mice were infected with BAA1717 and subsequently treated with different drug combinations for 72 hours. As shown in Figure 6A, the mice in the combination group were significantly protected from mortality compared with those in the other three groups, as the therapeutic effect was not ideal in the TFDG or cephalothin groups.

To investigate the pathological changes in the mice, the lungs were removed and sectioned in paraffin. As shown in Figure 6B, the lungs of the mice in the combination group appeared pink and soft, suggesting little damage to the tissue. However, in the other groups, the lungs were red and had a tight texture, suggesting severe hyperaemia. When observed under a microscope, the alveoli from the combination group were relatively intact, while the airways of the mice in the other groups were severely damaged by inflammatory cell infiltrates (Figure 6C). Furthermore, the quantity of bacteria colonized in the lungs of mice in the combination group was much lower than that observed in the other groups (Figure 6D), and lower lung wet‐dry ratios were observed in the combination group, indicating a lack of pneumonedema (Figure 6E). Taken together, the combination of TFDG and cephalothin systematically protected mice from *S aureus* pneumonia.

## 4 DISCUSSION

β‐Lactam antibiotics have been used for 70 years since their discovery and still account for approximately 65% of the total antibiotics used each year.^31^ However, the continuous abuse of antibacterial drugs has led to severe problems with drug resistance in many pathogens. The problem is particularly concerning for β‐lactam antibiotics, since they have been one of the most widely used antibiotics in clinical settings. β‐Lactamase secretion and the mutation of PBPs are the most important resistance mechanisms to β‐lactam antibiotics.^32^ However, PBP mutations may no longer be the major cause of induced resistance with the application of fifth‐generation cephalosporin, and the synergistic effect of β‐lactamase inhibitors and fifth‐generation cephalosporin is a potential therapeutic strategy against bacterial infections in the future.^33^ Fortunately, the usefulness of class A, C and D β‐lactamase inhibitors has been well verified, suggesting that the strategy of targeting β‐lactamase is effective in clinical treatment. However, the secretion of resistant MBLs is still a major issue owing to the presence of different active sites and substrate spectra in other β‐lactamases.

At present, most potential MBLs inhibitors reported in studies are metal chelators, such as mercaptotriazoles, phthalate and N‐arylsulphonyl hydrazine inhibitors.^34^ However, it remains unclear whether metal chelators have a negative effect on the normal biological functions of the host organism. For example, zinc plays an important role in nerve conduction, brain development, insulin secretion and other important life processes in animals, and the use of metal chelators as medicines may be potentially harmful to organisms.^35^, ^36^

In this study, BAA1717 was used as the experimental strain because MBLs are the only class of β‐lactamases that can be expressed by BAA1717. In the nitrocefin assay, four β‐lactamases with less than 5% identity were used as experimental proteins to screen inhibitors, and we observed that TFDG exhibited a strong ability to protect nitrocefin from hydrolysis by MBLs. The significant inhibition by TFDG of three different MBL variants indicates that TFDG is a broad‐spectrum inhibitor and provides an experimental basis for the application of TFDG as a potential MBL inhibitor in the future. We next determined the synergistic effect of TFDG with β‐lactam antibiotics to which bacterial strains have developed high levels of resistance, including penicillins (penicillin and ampicillin), first‐generation cephalosporins (cephalothin, cefazolin and cefradine), second‐generation cephalosporins (cefuroxime and cefaclor) and third‐generation cephalosporins (cefoperazone, ceftazidime and ceftriaxone), against BAA1717 by checkerboard and time‐kill assays. Although BAA1717 is a typical MRSA strain with complex PBP mutations, we observed that the sensitivity of MRSA to β‐lactam antibiotics could be partially restored following the inhibition of β‐lactamases. These results indicate the possibility of using a combination of β‐lactamase inhibitors and β‐lactam antibiotics against MRSA. In an MD simulation, Gln242 and Ser369 were observed to be important residues in the binding of TFDG to β‐lactamase N1, and the protective effect of TFDG in combination with β‐lactam antibiotics was further shown in a mouse *S aureus* pneumonia model.

Thus, targeting MBLs with TFDG may support the therapeutic effects of β‐lactam antibiotics, but this combined strategy still requires additional research. Finally, our results indicated that TFDG is a promising MBL inhibitor for use in combination with β‐lactam antibiotics against bacterial infections.

## CONFLICT OF INTEREST

The authors have no conflict of interest to declare.

## AUTHORS’ CONTRIBUTIONS

WJ, TZ, GY, LX, ZJ and NX conceived and performed all the experiments; TZ and YQ researched the data contributed to the statistical analysis and discussion; DX contributed to the discussion and reviewed the manuscript. All authors reviewed the manuscript.

## Data Availability

The data used to support the findings of this study are available from the corresponding author upon request.
